# Perception of animate motion in dogs

**DOI:** 10.3389/fpsyg.2024.1522489

**Published:** 2025-01-03

**Authors:** Judit Abdai

**Affiliations:** Center for Mind/Brain Sciences, University of Trento, Rovereto, Italy

**Keywords:** dog, animacy perception, biological motion, agency, animal-robot interaction, chasing

## Abstract

Various motion cues can lead to the perception of animacy, including (1) simple motion characteristics such as starting to move from rest, (2) motion patterns of interactions like chasing, or (3) the motion of point-lights representing the joints of a moving biological agent. Due to the relevance of dogs in comparative research and considering the large variability within the species, studying animacy perception in dogs can provide unique information about how selection for specific traits and individual-level (social) differences may shape social perception. Despite these advantages, only a few studies have investigated the phenomenon in dogs. In this mini-review, we discuss the current findings about how specific motion dynamics associated with animacy drive dogs' visual attention.

## 1 Introduction

**Figure 1:**
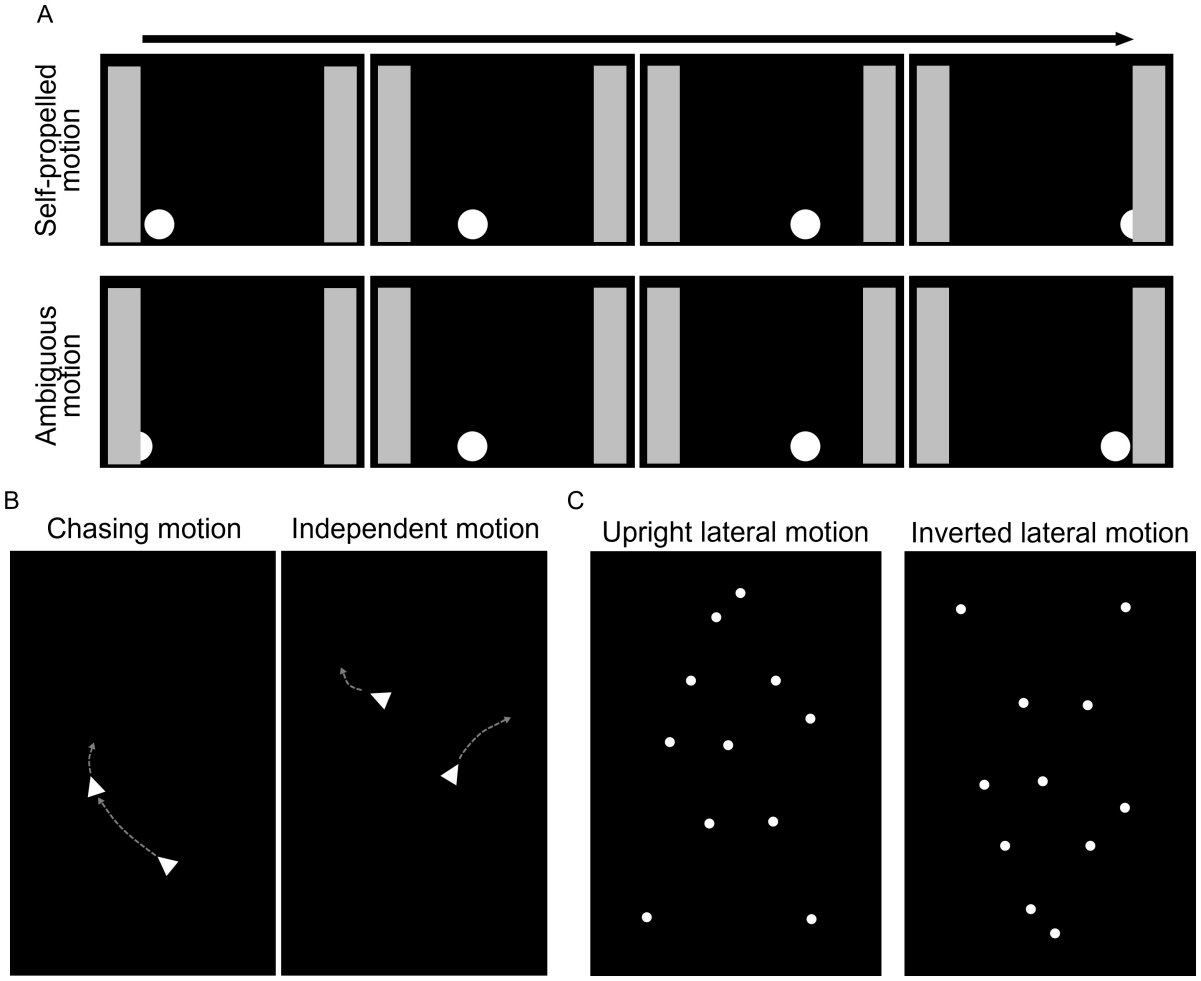
Examples of displays applied to investigate the perception of animate motion. **(A)** Schematic representation of the continuous motion of the dot. On the top, the onset of the dot's motion is visible and it moves out of view, disappearing behind the gray screen (self-propelled, animate), whereas on the right side the onset is ambiguous as the dot appears from behind the gray screen, and it stops when reaching the gray screen on the other side (ambiguous/inanimate motion). **(B)** On the left side, one dot is chasing the other, while on the right side they move independently from each other; gray arrows indicate the motion direction. **(C)** Point-light display of a laterally moving human figure, in an upright position on the left, and inverted position on the right side (figure based on Eatherington et al., 2019).

Attention to animate agents can facilitate to learn about the differences between animate and inanimate objects from birth, and later on it can also help to quickly detect predators, preys or social partners, and predict their future behavior (Scholl and Tremoulet, 2000; Lorenzi and Vallortigara, 2021; Schultz and Frith, 2022). Cues directing attention to such agents can be fairly simple, for example, two blobs on top and one in the bottom in the arrangement of eyes and mouth (face perception, for a recent review, see Kobylkov and Vallortigara, 2024) or the ability to initiate motion without external force (e.g., Premack, 1990; Mascalzoni et al., 2010; Di Giorgio et al., 2017). Some cues are more complex, either involving multiple objects representing a social interaction (e.g., chasing perception, Dittrich and Lea, 1994; Gao and Scholl, 2011; Frankenhuis et al., 2013; Meyerhoff et al., 2014; Atsumi and Nagasaka, 2015; Abdai et al., 2022a) or depicting the motion of a biological agent by point-lights representing the major joints of the body (biological motion, Johansson, 1973) (Figure 1). The phenomenon has been found in various species, including invertebrate species [e.g., human (Di Giorgio et al., 2021); dog (*Canis familiaris*) (Abdai et al., 2017b), chick (*Gallus gallus*) (Mascalzoni et al., 2010), common toad (*Bufo bufo*) (Ewert and Burghagen, 1979), zebrafish (*Danio rerio*) (Nunes et al., 2020), jumping spiders (*Menemerus semilimbatus*) (De Agrò et al., 2021)]. However, there are still a number of open questions about the evolutionary background, whether and how social and ecological environment influences animacy perception, and regarding potential changes in the perception (or behavioral response) during development.

Investigating animacy perception in dogs is advantageous because there is large variability within the species (e.g., selection for specific behavior traits), broad interindividual differences (e.g., sociability, training for specific tasks), and differences in social/ecological environment (e.g., pet dogs vs. free-ranging dogs) allowing the investigation of the influence of wide range of factors. Dogs are considered as an important model species to understand human social behavior (Miklósi and Topál, 2013). The possibility to compare their behavior to that of wolves with which they share a recent common ancestor but has evolved in a different environment in the past ca. 16–32,000 years (e.g., Kubinyi et al., 2007), or with other pet species (e.g., cats or miniature pigs; e.g., Marino and Colvin, 2015; Pongrácz and Lugosi, 2024) whose evolutionary and ecological histories, as well as the domestication processes differ, further highlights the relevance of dogs in comparative research. From a methodological point of view, it is also important that a wide variety of approaches can be easily applied for the investigation, which can provide us with a more complex overview of the phenomenon.

Although there is a significant interest in animacy perception in humans (Torabian and Grossman, 2023), research in dogs is scarce despite the advantages mentioned above. In the following, we will review the current findings of animacy perception in dogs. As research about static cues of animacy, such as face detection, role of fur and having filled rather than hollow insides (for a review, see Lorenzi and Vallortigara, 2021) is limited in dogs, we focus on dynamic cues, including (1) simple dynamic stimuli, (2) chasing pattern, and (3) biological motion.

Although animacy and agency are strongly related concepts, researchers have hypothesized that they are processed differently and thus should be treated distinctly (e.g., Spelke, 2000; Leslie, 2010). Animacy refers to the presence of some “life-like” features of the object, such as self-propulsion, whereas agency includes the agent having (some level of) control over its action, for example, moving in a goal-directed manner. Thus, in the case of displays of simple motion cues we refer to the acting object as “object,” whereas in the case of chasing and biological motion perception as “object/agent” as it is unclear which aspect of the motion might influence dogs' perception.

## 2 Main methodological approaches

In dogs, the phenomenon has been investigated by either using the video displays of specific stimuli, or by the live demonstration of motion patterns performed by artificial agents unfamiliar to dogs (Unidentified Moving Object, UMO). Applying video displays not only allows assessing the spontaneous visual interest/preference of subjects with highly controlled and reproducible stimuli, but by measuring pupil size changes further information can be obtained (Völter and Huber, 2022). Pupillometry in humans has been suggested to be a reliable measurement of arousal, attention and cognitive load (for a review, see Mathôt, 2018). Studies show that dogs' pupil size also increases when presented with angry emotional expressions (arousal; Somppi et al., 2017; Karl et al., 2020), and in the case of expectancy violation (Völter et al., 2023), providing a promising basis for animacy perception research.

The above methods give an insight about how specific motion characteristics can drive the attention and trigger the perception of animacy, but they do not provide information about whether and how it influences the subsequent behavior of dogs in relation to the observed object/agent (cf. Don't-Get-Caught task or wolfpack-effect in humans, Gao et al., 2009, 2010). Using robots to present the stimuli facilitates maintaining high control, replicability and reproducibility, and subjects can engage in physical interaction with the performing objects. Applying UMOs, that is, artificial agents capable of self-propelled motion and having an embodiment not resembling any animal species, allows to separate the influence of physical characteristics and motion on subjects' behavior. Flexible changes in the embodiment and motion features of the robot further contributes to the presentation of a wide range of stimuli (Abdai et al., 2018).

## 3 Perceiving animacy based on motion

### 3.1 Simple dynamic stimuli

One of the simplest motion cues that triggers animacy perception is self-propelledness, that is, the ability of an object to carry out (changes in) motion without visible external force (e.g., Premack, 1990; Leslie, 2010; Vallortigara, 2012; Schultz and Frith, 2022). These simple stimuli can include different motion characteristics, such as, initiating motion from rest (e.g., Mascalzoni et al., 2010; Di Giorgio et al., 2017), changes in speed (e.g., Rosa-Salva et al., 2016; Di Giorgio et al., 2021), change in the direction of motion (e.g., Tremoulet and Feldman, 2000), aligning the main axis of the bilateral body toward the direction of motion (e.g., Ewert and Burghagen, 1979; Hernik et al., 2014; Rosa-Salva et al., 2018; but see Rosa-Salva et al., 2023), or moving against gravity (Szego and Rutherford, 2008; Bliss et al., 2023).

In Völter and Huber (2022), dogs observed videos of events showing (1) objects being dropped by a human (inanimate) vs. the same event in reverse order, that is, the object initiating its own motion (animate); and (2) variability in the speed of an object (animate) vs. moving with constant speed (inanimate). Although regarding the looking time toward the events, they only found a difference between the animate and inanimate conditions in one instance, in all of the cases dogs' pupil size changed significantly during the presentation of the animate, but not the inanimate motion. Völter and Huber (2022) suggested that changes in pupil dilation in their study reflected an orienting response, balancing between visual sensitivity (dilated pupil) and acuity (constricted pupil) (see also Mathôt, 2018). In another study by Völter and Huber (2021) focusing on contact causality (Michotte, 1963), they further found that dogs' pupil size changed more and was overall larger when there was no contact between the two objects, that is, the second (“launched”) object started to move without a visible external cause. However, dogs looked longer at the “launched” object in the contact, and the “launching” object in the no-contact scenario (i.e., not at the object showing self-propulsion). These findings indicate that although overall looking time may not indicate preference, pupillometry may reveal sensitivity to animate motion cues in dogs.

Applying artificial agents (UMOs) (Abdai et al., 2022b), dogs were presented with the animate motion of a UMO including start-from-rest, visible acceleration and deceleration, and sharp change in direction; and with inanimate (ambiguous) motion, having the same dynamics of motion, but the key elements (e.g., moment of speed change) being occluded from the dogs. Subjects showed more interest toward the UMO that displayed animate motion, regarding both their looking behavior and physical contact with the UMO. Thus, it seems that simple visual cues lead dogs' attention to an object having animate motion characteristics, and facilitating dogs to enter into an interaction with these objects/agents.

### 3.2 Chasing perception

Simple motion dynamics provide a foundation for detecting animacy, but using more complex patterns may offer additional insights into the perception of animate entities. Chasing is an ecologically relevant pattern for many species, either in the context of predation (for both the predator and prey) or in social interaction (e.g., play). Several characteristics of the motion pattern can elicit the perception of the objects as animate, and parameters of the pattern are easy to manipulate, allowing to investigate the influence of different characteristics on the perception (e.g., Nahin, 2007; Scholl and Gao, 2013).

In a series of studies, Abdai and colleagues investigated chasing perception in dogs, by assessing dogs' looking duration toward geometric shapes displaying chasing pattern (dependent motion) vs. moving independently from each other, presented simultaneously on two sides of a screen. Both when using dots (Abdai et al., 2017b) and isosceles triangles (aligning their main axis with their motion direction) (Abdai et al., 2021) as moving shapes, dogs turned their visual attention to the independently moving figures shortly after the presentation started. Similar results were found in adult humans (Rochat et al., 1997; Abdai et al., 2017b, 2021) and 5-month-old human infants (Rochat et al., 1997). Such looking preference was suggested to be the result of the rapid perception of the chasing motion, which quickly led observers' attention to the independent motion, that is, the “unrecognized” pattern (for similar explanations in animacy perception, see Rochat et al., 1997; Kovács et al., 2016).

One interesting aspect of studying dogs' behavior lies in the large variability within the species. Selection for specific traits resulted in marked differences between breeds, including social behavior (e.g., Gácsi et al., 2009) and vision (e.g., distribution of ganglion cells in the retina and the visual field including; Peichl, 1992; McGreevy et al., 2004). When comparing chasing perception in hunting dogs (selected for chasing vs. retrieving), no overall difference was found between the two groups of dogs regarding their looking preference (Abdai and Miklósi, 2022). Thus, the basic mechanisms of animacy perception seem to be independent of the changes introduced by artificial selection in dogs.

Dogs were also presented with the live demonstration of chasing and independent motion patterns using UMOs (Abdai et al., 2017a). Following the observation of the UMOs' motion, subjects approached sooner the UMO that participated in the chasing interaction, also touching and grabbing sooner a ball attached to these UMOs after the demonstration. Thus, it seems that dogs were more likely to consider the UMOs from the chasing as potential interactive partners.

Although the above results indicate that dogs discriminate between a chasing and an independent motion pattern, it is unclear whether they indeed recognize the motion as chasing or reacted to another aspect of the motion (e.g., predictability, Lemaire et al., 2022). Although our findings showed that selection for specific behavior traits did not influence dogs' perception of the chasing motion (Abdai and Miklósi, 2022), between species comparisons may reveal how evolutionary background of the species or their socio-ecological environment influences their perception of the chasing motion. For example, predator and prey species may react differently or their perception is influenced by different motion characteristics. Also, within predator species, solitary vs. group hunting may influence perception of the moving object. For example, when using the video display of the chasing vs. independent stimuli, we previously found that although cats (*Felis catus*) also discriminate between the two patterns, they react differently than dogs (Abdai et al., 2022a). However, more information would be needed to reveal whether different behavior in cats was driven by differences in the perception of the pattern or other aspect of the stimuli or the method influenced their visual preference.

### 3.3 Biological motion

Applying chasing pattern facilitates the investigation of perceiving the interaction of multiple objects (dependency in the motion dynamics of two or more objects), but in animacy research it is also important whether stylized depiction of an animal's body can lead to its perception as biological motion and what information can be obtained from it. Despite the interest in the perception of biological motion in humans (for a recent review, see Troje and Chang, 2023), to date only five studies have investigated the phenomenon in dogs. In these studies, researchers presented the point-light displays (Johansson, 1973) of human or dog figures, that is, their regular social partners.

Eatherington et al. (2019) found that dogs preferentially looked at the motion of an upright dog figure compared to its inverted display, regardless of whether the point-lights representing the joints moved coherently or were scrambled. However, dogs did not react when the figure was a laterally moving human. The results of Delanoeije et al. (2020) were similar when presenting lateral motion of the human point-light figure, but applying a frontal moving human vs. an inverted-and-scrambled or just scrambled version of it, dogs preferred to look at the upright, coherent human motion. Thus, it seems that not only moving in accordance with gravity, but the global form of the figure is also important. Dogs reacting to the frontally but not to the laterally moving human figure indicates that spatial arrangement of the motion may be important for the perception. However, it cannot be excluded that their looking preference is not influenced by the (lack of) perception of the human figure but rather lateral motion is irrelevant from the viewpoint of interaction, resulting in lower visual interest (see also Ishikawa et al., 2018; Delanoeije et al., 2020).

Indeed, the results of Ishikawa et al. (2018) show that social relevance of the moving figure might influence dogs' perception of biological motion, or at least the behavioral response to the display. Frontal motion of a socially relevant agent (dog or human in this case) can be perceived as an initiation of interaction which can be positive for a highly sociable dog whereas taken negatively by a less sociable one. On the other hand, lateral motion can be of less interest if one seeks for social encounters, but may provide safer observation for an individual that prefers to avoid social interactions (Ishikawa et al., 2018). Their results were in line with this assumption, that is, dogs that scored higher on sociability toward humans looked less at the laterally moving human figure than those scoring lower. Dogs rated as highly social with other dogs also preferred to look at the frontal compared to lateral upright display of a dog, whereas those scoring low on sociability toward dogs showed the opposite looking preference (Ishikawa et al., 2018).

Ishikawa et al. (2018) relied on the general sociability of the dogs to see how it influences their perception of, and reaction to biological motion. Kovács et al. (2016) applied a different approach, in which they intranasally administered oxytocin to dogs (or placebo) that has been shown to increase social behavior toward other dogs and humans (e.g., Romero et al., 2014; Oliva et al., 2015). Oxytocin was found to increase sensitivity to biological motion in adult humans (Kéri and Benedek, 2009), but the results of Kovács et al. (2016) showed that although after receiving placebo, dogs looked longer at the biological than at the non-biological (inverted-and-scrambled) motion, this preference disappeared when they received oxytocin. Authors proposed that increased oxytocin might indeed facilitate the recognition of the biological motion in their subjects, but instead of focusing on this display, they rather directed their visual attention to the “unrecognized” stimulus (for similar explanation in chasing perception, see Abdai et al., 2017b).

Humans can obtain many information from point-light displays of a human figure, such as the gender of the figure (Mather and Murdoch, 1994), the action it performs (Manera et al., 2010), or the emotional state (Parkinson et al., 2017). Although dogs can find a hidden reward based on the pointing gesture of a human displayed on a screen (Péter et al., 2013; Eatherington et al., 2021), they did not follow the pointing when it was performed by a silhouette or a point-light display of a human (Eatherington et al., 2021).

Eatherington et al. (2021) suggested that dogs may react to the biological motion itself and do not recognize the displays as representing a human (or a dog), and based on the current findings, biological motion perception is not analogous in dogs and humans. However, aspects of the stimuli presentation beside animate motion might influence dogs' looking behavior (see below).

## 4 Conclusion and future directions

Although investigating animacy perception relying on looking preferences is a common approach in dogs (and humans), several factors other than animacy perception *per se* may influence dogs' looking behavior, either leading to the lack of, or opposite as (generally) expected preference (Kovács et al., 2016; Abdai et al., 2017b; Ishikawa et al., 2018). Dogs may be less motivated to watch two-dimensional displays on a screen as it is an artificial context for them, and it is also difficult to take into account all aspects specific to dogs' vision (e.g., differences in the visual field). As the results of Abdai and Miklósi (2022) suggests, differences in looking preference of dogs and humans may be influenced not by animacy perception, but rather by basic differences in the visual characteristics of the two species (e.g., dogs having slower and bigger saccades, and longer fixations than humans; see also Park et al., 2020). Also, the specific stimuli may be irrelevant for dogs (e.g., Ishikawa et al., 2018), or interest is influenced by another feature of the display (e.g., unfamiliarity) (Kovács et al., 2016; Abdai et al., 2017b). These can result in drawing false conclusions about the perceptual abilities of dogs. Relying on measurements other than looking preference, such as, changes in pupil dilation (e.g., Völter and Huber, 2021, 2022) can provide important insight about perception in dogs. Further, showing actions that are relevant for dogs or potentially leading to an interaction may also facilitate research on the topic (Abdai et al., 2017a, 2022b; Eatherington et al., 2021).

Research indicates that (1) simple motion cues associated with animacy influences dogs' perception of these objects/agents, (2) they rely on similar kinematic characteristics as other species, and (3) perception of an object as animate may provide a basis for dogs to establish further interaction with the agent. Still, we know little about, for example, (1) which cues may elicit such rich, spontaneous social perception, (2) how different animate cues may influence dogs' behavior toward an object/agent, (3) whether sensitivity to specific cues changes during development, (4) whether perceiving an object as animate leads to further expectations about its behavior (e.g., goal-directed motion; Biro and Leslie, 2007), and (5) about the neural mechanisms. Recent brain imaging studies in dogs investigated face- and/or body-sensitive (Bunford et al., 2020; Boch et al., 2023) brain areas, and neural representation of animate stimuli (human, dog, and/or cat pictures) vs. inanimate stimuli (Boch et al., 2023; Farkas et al., 2024); however, there is no information about the neural mechanisms underlying animacy perception (e.g., chasing or biological motion perception) in dogs. Applying neuroimaging and electrophysiological measures could provide meaningful contribution to comparative research on perceptual animacy.

Current data suggest that cats show preference to a UMO previously moving in an animate manner (Abdai et al., 2022b), they discriminate between chasing and independently moving motion patterns (although react differently than dogs in the same context) (Abdai et al., 2022a), and they prefer biological over non-biological motion (Blake, 1993). However, it is unclear which motion characteristics influence their perception, and whether and how different evolutionary and ecological background of cats and dogs might contribute to differences in their visual preference (see Abdai et al., 2022a). Comparison of dogs with other species (e.g., wolves or cats), and taking the large variability within the species, dogs may become important in testing the effect of a wide range of factors on animacy perception, including for example, selection for specific behavior traits (Abdai and Miklósi, 2022), individual differences (e.g., Ishikawa et al., 2018), or anatomy (see e.g., McGreevy et al., 2004; Bognár et al., 2018). Testing dogs also provide a unique opportunity to study how training for specific behaviors (e.g., hunting or herding), or different environment (e.g., pet vs. free-ranging dogs) may influence the perception. Future research in dogs may provide further insight about the evolutionary background and potential influence of environment on the perception of animacy or its influence on behavior.
